# An Instrumented Glove to Assess Manual Dexterity in Simulation-Based Neurosurgical Education

**DOI:** 10.3390/s17050988

**Published:** 2017-04-29

**Authors:** Juan Diego Lemos, Alher Mauricio Hernandez, Georges Soto-Romero

**Affiliations:** 1 Bioinstrumentation and Clinical Engineering Research Group—GIBIC, Bioengineering Department, Engineering Faculty, Universidad de Antioquia UdeA, Calle 70 No. 52-21, Medellín 050010, Colombia; mauricio.hernandez@udea.edu.co; 2 LAAS-CNRS, Université de Toulouse, CNRS, Toulouse 31400, France; gsotorom@laas.fr; 3 ISIFC, Université de Franche-Comté, Besançon 25000, France

**Keywords:** instrumented glove, wearable technology, hand dexterity assessment, IMU sensors, surgical simulation, movement signal processing

## Abstract

The traditional neurosurgical apprenticeship scheme includes the assessment of trainee’s manual skills carried out by experienced surgeons. However, the introduction of surgical simulation technology presents a new paradigm where residents can refine surgical techniques on a simulator before putting them into practice in real patients. Unfortunately, in this new scheme, an experienced surgeon will not always be available to evaluate trainee’s performance. For this reason, it is necessary to develop automatic mechanisms to estimate metrics for assessing manual dexterity in a quantitative way. Authors have proposed some hardware-software approaches to evaluate manual dexterity on surgical simulators. This paper presents IGlove, a wearable device that uses inertial sensors embedded on an elastic glove to capture hand movements. Metrics to assess manual dexterity are estimated from sensors signals using data processing and information analysis algorithms. It has been designed to be used with a neurosurgical simulator called Daubara NS Trainer, but can be easily adapted to another benchtop- and manikin-based medical simulators. The system was tested with a sample of 14 volunteers who performed a test that was designed to simultaneously evaluate their fine motor skills and the IGlove’s functionalities. Metrics obtained by each of the participants are presented as results in this work; it is also shown how these metrics are used to automatically evaluate the level of manual dexterity of each volunteer.

## 1. Introduction

Fine Motor Skills (FMSs) or dexterity is the coordination of small muscles, in movements—involving the synchronization of wrists, hands, and fingers—with the eyes. FMSs are the set of abilities that humans use when performing complex actions such as picking up small objects, writing, painting or sewing [1,2]. FMSs appear at the moment of birth; in childhood, motor skills continue to evolve, particularly during school age. Refinement of motor skills in adults is strongly influenced by the daily activities they carry out. Motor skills decrease significantly as a result of physical and cognitive deteriorations caused by aging [3].

A high degree of FMS is an essential requirement for anyone who wants to become a surgeon; this is much more significant in medical specialties such as neurosurgery, as small errors may imply severe consequences for the patient. FMS is related to the deftness with which surgical instruments are manipulated, the gentleness while handling tissues, the time and amount of movement required to perform each task, the degree of precision for reaching target areas, and the amount of force applied [4,5]. Relatively few studies have been conducted to find out key aspects to be evaluated in future neurosurgeons [6,7]. However, there is an agreement around the relevance of evaluating hand dexterity, because it allows a more efficient technique and greater ability to achieve success in surgical procedures [8,9]. Assessment of this parameter has often been subject to criteria of instructors, or at its best, to the review by a panel of experienced surgeons who examine video recordings from operations performed by residents [5]. The use of surgical simulators as training tools can be considered as a complement to the traditional master-apprenticeship approach. In those mock procedures, trainees are asked to repeat simulation exercises on “simulated patients”, until they have learned the required skills to deal with “real patients” [10]. As a consequence of the situations mentioned above, future surgeons will improve their abilities insofar as they spend time in front of a simulator (most of the time, there will be no experts nearby to help). Therefore it is necessary to implement tools to enable an efficient assessment of manual dexterity in surgical simulations.

Formerly, simulators were focused only on training in specific tasks; this technology has now evolved. Thus new advanced simulators are not only training systems, but they can be assessment tools at the same time [11]. For their use in FMS assessment, surgical simulators should include a combination of hardware and software that enables to measure and process information from variables such as hand movements, applied forces, trajectories and timing [5,9,12,13,14,15,16]. These features are now available in most advanced devices like LapSim and LapVR, for laparoscopic surgery or NeuroVR, for neurosurgical simulation [17,18,19]. Those simulators use haptic peripherals to track hand movements and also recreate the sense of touch on Virtual Reality (VR) environments [18,19]. However, most haptic meaning-based applications are expensive to develop and requires a big research team, since they often entail significant engineering challenges and the use of advanced computational models [9,20]. Modern VR simulators still need to be improved, to match real models in aspects like tissue behavior, tactile sensation, and anatomical appearance [21]. Some of these limitations can be partly attributed to the small ecosystem of haptic peripherals available, so, the range of possible applications for the simulation of surgical instruments is often limited to simple pen-like or scissors-like devices [22,23,24]. Alternatively, traditional bench top and manikin-based simulators use physical models that emulate anatomical structures. Those models are quite realistic, giving the trainee the impression of working with “real things”. Also, they are made with materials that resemble real tissues, making touch sensations lifelike [11]. Although this technology has been used in medical education for more than fifty years, it still lacks tools for hand tracking or manual dexterity assessment.

To improve the capabilities of traditional simulators, many custom hardware/software designs useful for hand motion analysis and surgical dexterity assessment have been developed; they can be categorized to non-glove and glove-types. Datta et al. [25,26] used an electromagnetic tracker as a motion analysis system that measures the number of hand movements made and the time taken to complete each task and found that hand motion analysis may be an effective objective measure of dexterity in open surgical simulation. Ross et al. developed a semi-automated catheter-tracking software from motion analysis of fluoroscopic videos, and they found that it is a valuable tool for the objective assessment of endovascular skills [27]. Solutions that used image processing are based on cameras which use the visible or infrared spectrum [28]; some of them, take direct pictures of the hand [29,30], while others rely on visual detection of optical markers in the hand [31]. In general, they are accurate but require line-of-sight (a straight path without obstacles between cameras and tracked objects), and as an additional drawback, they are affected by lighting, so they are only useful in environments with well-controlled conditions. Zappella et al., Gray et al., and Uemura et al. used digital video systems to capture hand and instruments movements of surgical trainees, information is processed and used to perform surgical gesture classification and assess laparoscopic skills objectively [32,33,34]. Harada et al. designed instrumented tweezers with the ability to measure several parameters of instrument manipulation during anastomosis simulations, hardware include an Infrared optical motion tracking system, IMUs, and strain gauges [35]. Hammond et al. designed a similar device, using an electromagnetic tracker, and also they presented a novel sensor technique to print metallic strain gauges on the surface of surgical instruments [36,37]. Zihni et al., and Overby and Watson used IMU sensors to register hand movements and instrument trajectories. Acquired data were numerically manipulated using different digital signal processing approaches to produce metrics to assess FMS [38,39,40]. Finally, electromyographic (EMG) signals have been used for hand movement processing, they can appropriately recognize basic hand gestures, but they are useless for fine finger movements analysis [41]. Additionally, EMG has the disadvantage of requiring the installation of surface electrodes [42], which usually are uncomfortable during training, and require some level of expertise to make sure that electrodes are correctly installed.

Glove solutions or Instrumented Gloves (IGs) are wearable systems that have been used in hand movement analysis for years; this is the reason why there are many IGs available on the market [43,44,45]. Although those IGs are now a relatively mature technology, they are in general too bulky to be used comfortably in medical simulation. Many of these IGs that can be found in the literature are designed as tools for the treatment of people who has Parkinson’s disease or suffered a stroke [46,47,48]. IGs equipped with optical encoders, or fiber-optic nerves have been developed [49,50]. This technology made it possible to produce IGs that fit different hand sizes, and they also bring a good movement freedom, but they can only record the movement of fingers with respect to the hand (they cannot provide information about hand movement). Something similar happens with IG based on bend sensors, they commonly use force sensitive resistors used as flex sensors to measure movements of each finger [51,52]. Therefore, there is no data associated with the hand itself. IGs based on electromagnetic sensors can detect the movements of fingers and hand [15,53]. However, this kind of systems requires robust computational processing to reduce the susceptibility to electromagnetic interference generated by other electric devices and metal objects [54]. Finally, IGs based on Inertial Movement Units (IMU) have recently emerged as a powerful alternative [55,56]. IMUs are quite small, as they can be unobtrusively embedded on wearable designs [57], this avoids using bulky and loose parts on IGs. The benefits of these IGs are plentiful; they can monitor the movement of the hands and fingers, they do not require line of sight, and they are not affected by electromagnetic interference.

The aim of this paper is to present the IGlove system, a wearable device which is composed of an IG that uses IMUs to capture hand movements and a set of software applications that process IMUs signals to estimate some of the metrics that are commonly used by modern surgical simulators to assess trainee’s manual dexterity. IGlove was developed in cooperation between GIBIC group in Colombia and S4M group in France [58,59]. The main objective of this paper is to show how to process acquired data to obtain hand dexterity metrics that are suitable to assess FMS on surgical simulation environments automatically. IGlove was originally conceived to be integrated to Daubara NS Trainer (a neurosurgical simulator previously developed by GIBIC group), but it could be easily integrated to another manikin- or benchtop-based medical simulators, or even in other applications where hand motion analyses are required.

The rest of the paper is divided as follows: Section 2.1 provides a detailed description of the IGlove’s hardware architecture. Section 2.2 shows the details regarding the design of a test for assessing FMS of a group of volunteers using IGlove, in which each volunteer was filmed while he was doing the test. Section 2.3 shows how FMSs were evaluated “manually”, according to the criteria of human evaluators. Section 2.4 describes algorithms utilized to obtain manual dexterity metrics. Section 2.5 makes a dimension reduction analysis, in order to assess the real necessity of using all the data acquired by each sensor. If it is found that the analysis can be carried out using fewer signals or sensors, the complexity of the problem may be significantly reduced. Section 2.6 presents some statistical tools to classify volunteers according to their level of FMS based on the previously-mentioned metrics. Section 3 shows the results of IGlove’s test, which were obtained from the methodological design proposed in Section 2. Section 4 presents a discussion of the results and their applications in neurosurgical education. Finally, conclusions and future trends are presented in Section 5.

## 2. Materials and Methods

### 2.1. IGlove Description

**Figure 1:**
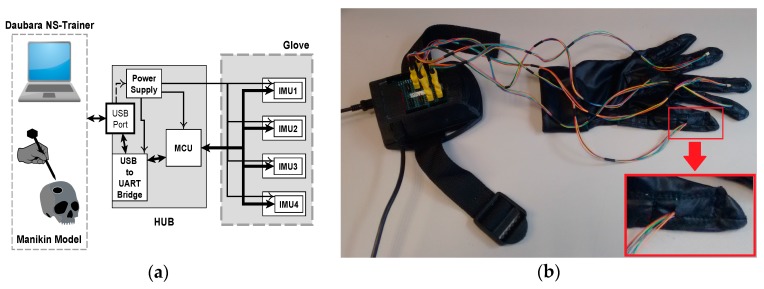
Hardware of IGlove system: (**a**) Block diagram of main parts of Hub and Glove and their interconnection. Microcontroller and sensors communicate in a single-master, multiple-slave configuration using SPI on a multidrop bus. IMU sensors were configured to sample internal signals from accelerometers and gyroscopes at 8 KHz, and the user-programmable digital low-pass filters were setting up to a cut-off frequency of 125 Hz, using the preconfigured low-pass filters of the sensor. Measurement data is sent via a high-speed USB link to the Daubara NS Trainer; sensors are sampled 100 times per second; (**b**) Picture of the system showing the connection between Glove and Hub. In this case, sensors were worn on thumb, index, middle, ring fingers, and in the back of the hand. A communication system that transfers data and power between Hub and each sensor has been implemented using a set of thin and flexible cables. A zoom of the thumb is presented in the lower right corner; it can be observed how power and data cables enter to one sensor compartment.

The IGlove hardware consists of two main parts: the Glove and the Hub (Figure 1a). The Hub’s architecture is based on an electronic board that features a Cypress PSoC4 CY8C4245AXI-483 as the core of this system; this ARM Cortex M0 microcontroller is responsible for setting up an SPI interface with IMU sensors on Glove [60]. The Hub features a power supply based on an LDO regulator that converts 5 V from USB port to 3.3 V to feed the circuit. Acquired data is transmitted to the Daubara NS Trainer using a USB to serial UART bridge; processing algorithms will be discussed in Section 2.4.

**Figure 2:**
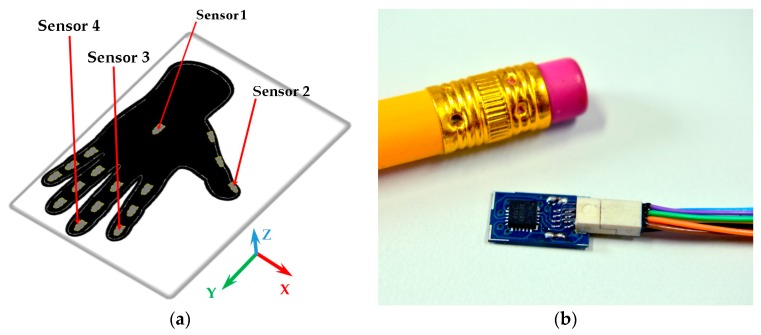
Sensor compartments and a motion sensor: (**a**) Distribution of sensor compartments on IGlove. Red arrows point to compartments used to accommodate the four sensors employed in this study; Sensor 1 is in the back of the hand, Sensor 2 on the thumb, Sensor 3 on the index, and Sensor 4 on the middle finger. 16 gray marks on glove indicate available places to put motion sensors (The glove has 16 compartments to put sensors, three for each finger on each phalange, and one in the back of the hand); (**b**) A sensor beside a 6 mm pencil to illustrate size. This sensor finely fits on any of the compartments on the Glove.

The electronics are housed in an ergonomic custom-designed enclosure. The Hub is secured to user’s arm by using an adjustable strap (see Figure 1b). For the glove fabrication, several fabrics were tested, a thin and elastic fabric was chosen to ensure users comfort during exercises. To warranty hygiene and cleanliness gloves are washable. Also, there is a glove for each user. The glove comes in two sizes, large and small (especially useful for people with small hands). IGlove’s electronics do not have direct electrical contact with skin and has been manufactured with materials that are inert to the skin. Therefore, this wearable design does not represent drawbacks of biocompatibility or electrical safety. IMU Sensors are installed in specially designed compartments (small pockets) on the glove, where sensors can be precisely adjusted, as shown in Figure 2a. IGlove’s sensors can be easily removed from the glove; then they can be used with the next user. This feature allows customizing the sensor distribution to perform different types of studies, just by placing sensors in various parts of the hand. Current Hub configuration permits the connection of up to eight sensors, but it can be easily extended.

In order to determine the appropriate number of sensors to be placed in the glove, a group of neurosurgeons and residents of neurosurgery was observed while handling some surgical instruments like biopsy needles, dissectors, and ultrasonic aspirator. It was found that during manipulation of those instruments, specialists preponderantly used only three fingers, thumb, index and middle finger; ring finger and little finger were rarely used. For these reasons, it was decided to embed four sensors on IGlove, on distal phalanges of fingers commonly used for instrument handling (thumb, index, and middle) and on the back of the hand. However, the volume of data from those four sensors (which generate 36 signals in total), translates into a huge challenge regarding analysis and processing of information. On Section 2.5 a procedure which identifies sensors and signals that are more relevant for FMS assessment is presented. Thus, the redundant and irrelevant information can be ignored, significantly reducing the complexity of the problem.

A sample of each sensor is shown in Figure 2b. It is based on MPU-9250 from InvenSense, which is a multi-chip module consisting of two dies integrated into a single package. One die houses the 3-axis gyroscope and the 3-axis accelerometer. The other die houses a 3-axis magnetometer. Hence, the MPU-9250 is a truly 9-Degrees of Freedom (DOF) motion tracking device in a 3 × 3 × 1 mm QFN package [61]. A little circuit board was designed to mount the IMU, due to the severe size constraints imposed by the small sensor compartments. Special considerations regarding connectors and communication lines that connect sensors to hub were taken. As cables will be draped over the user, those cables should slide freely without tangling with instruments used in the simulation. Cables must also bend, twist, and flex without impeding hand movements. A little 8-pin connector was used to bring power lines (GND and +3.3 V) and communications (four-line SPI and interrupt) from the hub. For those purposes, 0.25” pitch Wired Polarized Nano Connectors from Omnetics were used, they are small enough and incorporate exceptionally thin and flexible cables [62].

### 2.2. Test Design

A total of 14 volunteers were recruited to perform a test for evaluating IGlove’s functionalities. Ten trainees were male, and four were females, age range between 23 to 32 years old, all of them were right-handed, and none of them had previous contact with medical simulators or psychometric tests for measuring hand dexterity. They manifested had not consumed drugs or alcohol in the past two days, and they are not suffering from any illness or condition that would prevent them from doing the test. All volunteers were informed in detail about the study, and all of them signed an informed consent for participating in this study. The test consisted of three exercises to be performed while they wore the IGlove. Between exercises, there were periods of rest, during these periods the participant was asked to put his/her hand on the table in a neutral position. Rest periods were included with a dual purpose, first, give a break to the volunteer, preventing fatigue to affect his/her performance, and secondly, as it will be shown below, to facilitate exercises segmentation.

**Figure 3:**
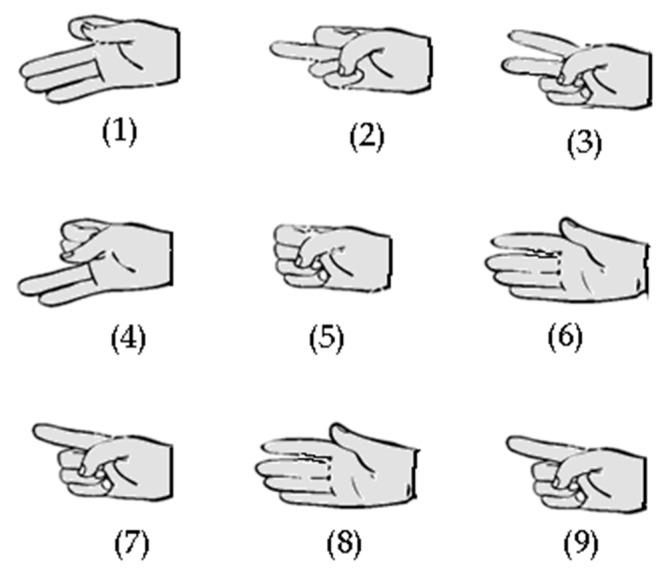
Hand gestures (1 to 9) used in exercises one and two. Those gestures were designed to test the capability of IGlove system for recognizing finger movements, using sensors on the thumb, index, and middle fingers.

The first exercise prompted the participants to run the group of hand gestures shown in Figure 3; after each gesture, the participant must put his/her hand in the rest position for a few seconds. These gestures were designed to verify the ability of IGlove to register fine finger movements; rest periods were used to simplify movement recognition process. In the second exercise, the participant had to run the same sequence, but this time, he/she performed the entire sequence without putting his/her hand in the rest position until finished. This exercise was designed to assess the ability of the system to recognize movements without the help offered by rest periods.

**Figure 4:**
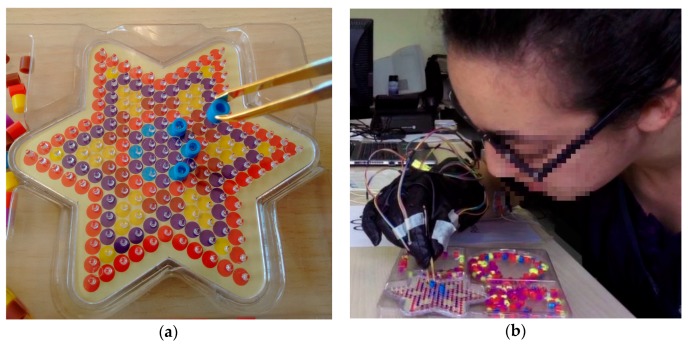
The third exercise of IGlove test: (**a**) Shows the PegBoard, tweezers holding a plastic bushing (PB) and a portion of PBs deposits on the far right of the image. The PegBoard features many pegs, but subjects only need to put six blue PBs on blue pins and one red PB on each peak of the star; (**b**) A picture of one of the volunteers while running the third exercise (she is just placing a blue PB on a blue peg).

The third exercise of the test was focused on FMS assessment. It was designed based on traditional Neuropsychological Tests (NT), which have been concerned with the objective measurement of skills in psychometrics. PegBoard Tests (PBT), like Grooved PegBoard and Purdue PegBoard, are NTs that have been specifically designed as tools for FMS assessment, but commonly they are only used to evaluate motor or cognitive impairment in patients and for personnel selection purposes [63,64]. However, some authors have explored the possibility of using this type of NTs to assess dexterity in surgeons [7]. Researchers have found that there is a significant relationship between scores on NTs that evaluate FMS and the performance of trainees in surgical procedures [65,66,67]. Moreover, Kyle et al. [68] suggest that NTs may be used to identify those novice trainees with lower FMS scores, who might require additional support for their learning of some specific technical tasks. In designing the third exercise, it was also taken into account that most of the laparoscopic simulators include PBT-like modules called Peg Transfer Boards [69]. Those PBT-like modules are used to assess and improve FMS by requiring the surgical trainee to transfer small objects between designated pegs using laparoscopic instruments [67,70]. The third exercise of the test was also designed as a PBT-like module. A star-shaped pegboard was used, featuring many pegs and three deposits for small multicolored Plastic Bushings (PBs), as shown in Figure 4a. Volunteers were given the task of moving six blue PBs from deposits to blue pegs, and in the same way, six red PBs to the vertices of the star (six red pegs), employing, for this purpose small tweezers. Figure 4b presents a volunteer of the test while running this exercise. This kind of exercises was selected due to its ease of implementation, and its extensive use as a tool for assessment FMS which ensures its validity [70]. Trainees did the test only one time because the idea was to use the third exercise as a psychometric test to measure their current level of dexterity. That is the reason why they were asked if they had ever made a test like this one before.

### 2.3. Video Review of Test

All volunteers were filmed while performing the test, much more attention was given to the third exercise, as it was specifically designed to assess FMS. Videos were subsequently used to achieve two objectives, first, to ensure that exercises were correctly performed, and second, to perform an assessment of the test through a careful examination of video records (human-aided assessment). As described above, the third exercise involves to take a PB from one deposit and place it into the corresponding peg; this action was repeated twelve times. Each repetition of the exercise was segmented into two basic periods of time: firstly, *Task1*, which starts when a PB is picked up from one deposit using the tweezers and placed onto the appropriate peg (it is the action of transporting the PB). Secondly, *Task2*, which consists of the remaining shares that must be done just before picking up the next PB. In some cases, *Task2* may not only represent the amount of time required to return to pick up another PB, since participants often made mistakes, performed unnecessary movements, and wasted time in trying to find out the easiest PB to pick up from deposits. Time wasted on making these actions was added to *Task2* as a penalty mechanism. Some of the actions considered as errors include: drop a PB, misplace a PB or place it in a wrong place, try to use the other hand, perform any sudden or unnecessary movements, and fail to pick up a PB from a deposit.

At the end of the exercise *T_Span_*, *Total_Task1_*, and *Errors* parameters were calculated. *T_Span_* indicates how long was the exercise (sum of *Task1s* and *Task2s*), *Total_Task1_* is calculated from the from the sum of time invested by each participant for transporting PBs (sum of *Task1*), *Errors* is the number of errors during the exercise according to the criteria explained in the previous paragraph. Under these assumptions, people with better FMS should get lower values of *T_Span_*, *Total_Task1_*, and *Errors* as they can do the exercises quickly and make fewer mistakes. Parameter *T_Ratio_* is also calculated as *T_Ratio_* = *T_Span_/Total_Task1_*; it is an indicator of efficiency in the exercise; participants who did the exercise efficiently, obtained low *T_Ratio_* values, as they employed most of the time to transport PBs. Conversely, actions such as taking too long to pick up a PB, make mistakes, correct previous errors or make unnecessary movements, cause an increase in *T_Ratio_*, indicating lower performance.

A clustering analysis was made based on the information gathered from video review; it was used to classify individuals according to the demonstrated abilities in the third exercise. The standardized Euclidean distance was used as a metric to measure dissimilarity on data. Ward’s linkage was used for clustering.

### 2.4. IGlove Data Processing

**Figure 5:**
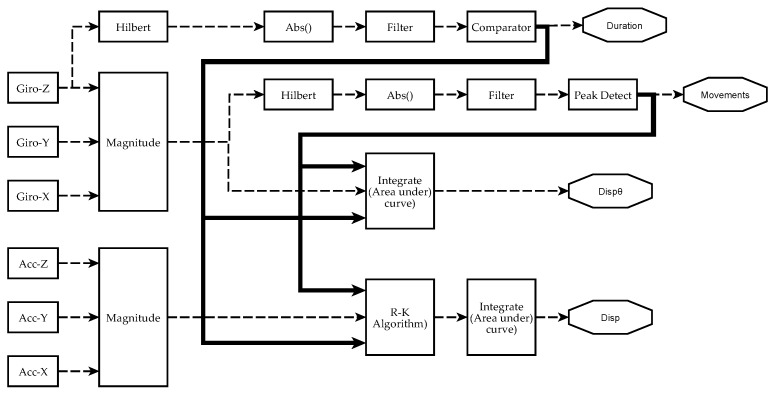
Scheme of signal processing and information analysis to obtain manual dexterity metrics on each sensor. Currently, four metrics have been achieved from IGlove’s raw signals: Duration of exercises (*Duration*), the number of movements during each exercise (*Movements*), spatial displacement (*Disp*), and angular displacement ($Disp_{\theta }$). The absolute value of the Hilbert transform of a gyroscope signal is filtered to obtain a smoothened signal that is higher than 0 when the hand moves. A comparator decides when the level and duration of a perturbation in the signal were enough to mark it as an exercise. The metric *Duration* of an exercise is the difference between the end and the beginning of a significant perturbation. Variable *Movements* is calculated from the magnitude of angular speed $S_{\omega }$ (computed from all gyroscopes using Equation (1)). It was observed that peaks on $S_{\omega }$ indicate when a movement was performed. To estimate the number of moves, $S_{\omega }$ is smoothened, but this time, a peak detector identifies the spikes in the signal that were large enough and have been caused by a valid hand movement. The area under $S_{\omega }$ was calculated using numerical integration to estimate $Disp_{\theta }$. Spatial kinematic parameters were estimated from accelerometers signal $S_{a}$ (calculated using Equation (3)), Runge–Kutta method was used to obtain an approximate time series for the magnitude of velocity $V_{a}$. Finally, spatial displacement is calculated as the area under the curve of $\left|V_{a}\left(t\right)\right|$. When performing numerical integration, Duration and Movements parameters were used to establish integration limits and estimate initial conditions.

The data processing scheme to obtain metrics from each sensor is briefly depicted in Figure 5. An initial pre-calibration process was carried out to eliminate offset errors on sensor measurements. Section 2.4.1, Section 2.4.2 and Section 2.4.3 give detailed descriptions of signal processing and information analysis performed to obtain manual dexterity metrics. It is worth to mention that the system can calculate absolute orientation angles using sensor fusion algorithms [71]. However, this feature is not shown in Figure 5, since these angles were not utilized in the analysis described in this paper (as shown in Section 2.5 and Section 3.3).

#### 2.4.1. Exercises Segmentation (Estimating Metric Duration)

The first analysis was focused on developing a mechanism to automatically determine the duration of exercises, i.e., an algorithm for detecting the beginning ($t_{ini}$) and the end ($t_{end}$) of each of the exercises. To facilitate this process, volunteers were asked to put right hand in a resting position on the table for several seconds at the end of each of the exercises. Thus, time periods of activity corresponded to those where sensors detected a significant amount of movement [72].

#### 2.4.2. Movements Segmentation (Estimating Metric Movements)

The second analysis was focused on estimating the number of movements carried out within exercises. For this purpose, the magnitude of angular speed ($S_{\omega }$) was calculated from the X, Y and Z gyroscopic signals using Equation (1):

(1) $$S_{\omega }=\sqrt[{2}]{\omega_{x}^{2}+\omega_{y}^{2}+\omega_{z}^{2}}$$

**Figure 6:**
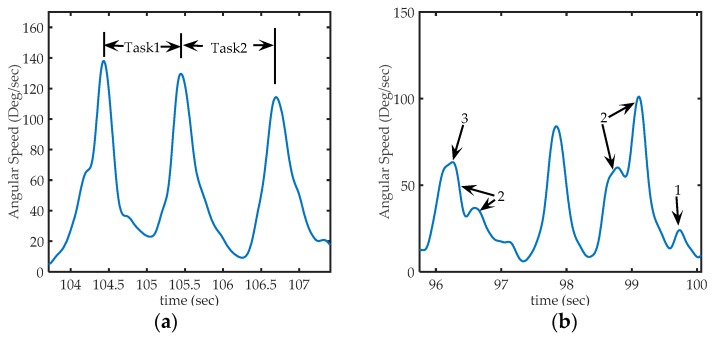
Sections of $S_{\omega }$ acquired from sensor 1 (in the back of the hand), (**a**) Representation of peaks on $S_{\omega }$ signal on third exercise: The first peak corresponds to the time where a PB is collected from deposit, the second peak, corresponding to the instant at which the PB is placed in the corresponding peg and, the third peak corresponds to the action of taking the next PB. *Task1* occurred between the first and second peak, and *Task2* occurred between the second and third peak; (**b**) Some particular cases of peaks that can be found in the $S_{\omega }$ signal. Arrow 1: shows a peak too small to be considered a voluntary movement (to be discarded), Arrows 2: shows sets of nearby peaks that represent single movements, and arrow 3, shows a much smaller peak, but not small enough to be eliminated (it represents a hand movement).

$S_{\omega }$ signal was used to discriminate individual hand movements, as it was observed that peaks on $S_{\omega }$ signal occurs each time the hand movement changes (e.g., a change between Task_1 and Task_2) [73]. Therefore, those peaks were used to segment the movements in epochs. Figure 6a shows a segment of a typical $S_{\omega }$ signal in the third exercise. The first peak corresponds to the moment where a PB is collected. The interval linking this first peak with the second corresponds to the transport of a PB to the board. The second peak corresponds to the time in which the PB is put on the peg. The interval linking the second peak with the third corresponds to the return to the reservoir. Finally, the last peak corresponds to the action of taking the next PB. It was also found that only some peaks on $S_{\omega }$ were well shaped as those shown in Figure 6a. Often, it was common to find peaks as shown in Figure 6b. Many of them with a different shape (high frequency), others too small to be considered a consequence of voluntary movements, and were probably caused by small motion artifacts or even can be attributed to errors in measurement. In some cases, sets of peaks (groups of peaks very close to each other) appear while the volunteer performed the movement. This kind of events are related with variations in the rotation speed while performing a hand movement, a particular way to do the exercise.

An algorithm to eliminate artifact spikes from $S_{\omega }$ signal was developed. Firstly, $\left|\hat{S_{\omega }}\right|$ the absolute value of the Hilbert’s transform of $S_{\omega }$ is calculated. Secondly, resulting signal is smoothed by a FIR filter, using a sliding Hanning window filter (window length = 200 ms). Finally, a peak detection algorithm removes all those cases of peaks that cannot be attributed to hand movements (as it was shown in Figure 6b), the algorithm skips those spikes that are too small to be considered voluntary movements and treats peaks very close to each other as a single movement. Movements performed during an exercise are represented as time intervals between each pair of remaining peaks. This motion sensing technique can also detect erroneous movements. If the amount of movements required to complete an exercise is known, then, additional peaks will evidence the presence of errors or unnecessary movements. Even if the “ideal” amount of movements is unknown, it is also possible to make comparisons with the amount of movements required by a very skilled individual. On surgical simulators, a parameter called movement economy is used; it is calculated as a ratio between the average of movements required by skilled surgeons (taken as a gold standard) and the average of movements performed by a trainee [65].

#### 2.4.3. Kinematic Analysis

Surgical simulators use metrics based on the angular and spatial displacement; it has been found that the most skilled users and experienced surgeons perform much shorter paths in simulations [74]. From the numerical integration of the signals from the accelerometers and gyroscopes, estimation of the spatial and angular displacements can be obtained using IGlove system.

The area under the curve of $S_{\omega }$ was estimated to obtain a measurement of angular displacement during an exercise ($Disp_{\theta }$). $S_{\omega }$ Signal was previously detrended to reduce gyroscope error issues:

(2) $$Disp_{\theta }=\int_{t_{ini}}^{t_{end}}\;S_{\omega }dt$$

The magnitude of acceleration signal $S_{a}$ was calculated from the X, Y and Z components of acceleration and subtracting the magnitude of the gravitational acceleration (*g*):

(3) $$S_{a}=\sqrt[{2}]{a_{x}^{2}+a_{y}^{2}+a_{z}^{2}}-g$$

$V_{a}\left(t\right)$ which is the function of speed magnitude can be obtained by solving the Equation (4):

(4) $$\frac{dV_{a}\left(t\right)}{dt}=S_{a}\left(t\right)$$

To solve Equation (4) Runge-Kutta method was used. Numerical integration was performed for each hand movement, i.e., between each pair of consecutive peaks of $S_{\omega }$. The initial condition for $V_{a}$ on each movement was the speed calculated at the end of the previous movement; the first initial condition $V_{a}\left(t_{ini}\right)=0$, as the hand was in a rest position at the beginning of the exercise. Performing speed calculations for each of the movements and not for the whole exercise reflects hand dynamics better and contributes to reducing offset errors of numerical integration.

Calculation of total absolute displacement during an exercise (Disp), can be numerically calculated as the area under the curve of the absolute value of the signal $V_{a}\left(t\right),$ the numerical integration was performed using the trapezoidal technique. The absolute value is necessary, otherwise, the value of the total displacement would be zero:

(5) $$Disp=\int_{t_{ini}}^{t_{end}}\left|V_{a}\left(t\right)\right|dt$$

### 2.5. Dimension Reduction

Dimension reduction analyses were carried out to evaluate the real necessity of using all the data acquired by each sensor on IGlove, with the objective of determining if the hand motion assessment can be carried out using fewer signals and sensors, if this can be done, the complexity of the problem may be significantly reduced. The correlation coefficient (r) was calculated between signals from the four sensors, with the premise that if a high *r* between two sensors is found, one of them is redundant (so, one of them is not considered for the analysis). Additionally, another analysis was conducted, focusing on whether or not it is necessary to integrate the signal from 3-Axial magnetometers. Although, those sensors are not easy to use, since they require calibration to deal with Hard- and Soft-Iron interference [75], Earth’s magnetic field measurements can be used together with data from accelerometers and gyroscopes to determine the absolute orientation angles (ϕ, θ and ψ), by using fusion algorithms [71]. Approximations of angular speeds on each axis can be calculated by approximate derivatives:

(6) $$\omega_{\phi }=\frac{d\phi }{dt}\;\omega_{\theta }=\frac{d\theta }{dt}\;\omega_{\psi }=\frac{d\psi }{dt}$$

Similarly, to Equation (1), the magnitude of angular speed calculated from fusion algorithms ($SF_{\omega }$) can be calculated using Equation (7):

(7) $$SF_{\omega }=\sqrt[{2}]{\omega_{\phi }^{2}+\omega_{\theta }^{2}+\omega_{\psi }^{2}}$$

A comparison between results obtained with Equation (1) (that used data measured by accelerometers) and Equation (7) (that used data estimated from fusion algorithm) was performed to see whether these magnetometer data were relevant or not.

### 2.6. Statistical Analysis

From the results above, a clustering of all subjects who participated as volunteers in the test was performed. Metrics *Duration*, *Movements*, $Disp_{\theta }$, and Disp, were used as variables for this purpose. Once again, the standardized Euclidean distance was used to measure dissimilarity and Ward’s, algorithm was used for clustering. Results of this automatic classification were compared with the results previously obtained from video review. Additionally, a nonparametric test (Kruskal-Wallis, an alternative to the ANOVA test), was used to assess the validity of the classification obtained from clustering. The selection of the nonparametric test was based on the small size of our sample and the stochastic heterogeneity of the samples. The purpose of this test was to evaluate that each one of the variables has significant different distributions across both groups (various dexterity levels). The significance level of the Kruskal-Wallis test was set to 1%, and the comparison was performed using the Bonferroni method for correction.

## 3. Results

### 3.1. Results from Video Review

**Figure 7:**
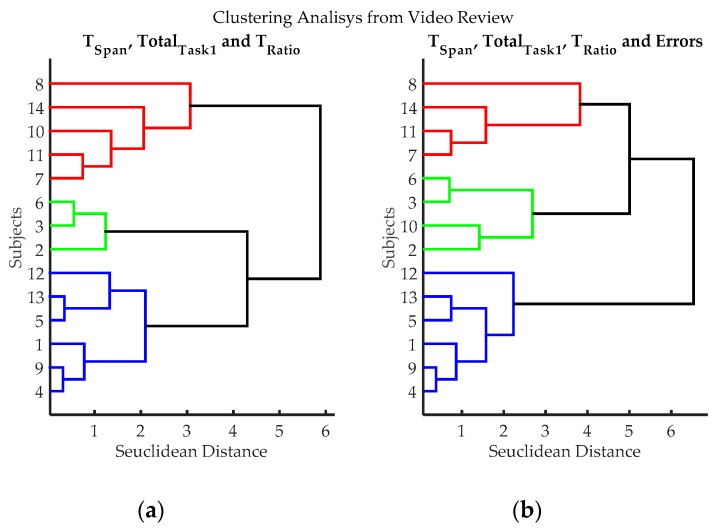
Clustering results according to the variables evaluated from video records of volunteers while executing exercise 3. (**a**) Clustering classification using variables: *T_Span_*, *Total_Task1_*, and *T_Ratio_*. *Errors* were not utilized in this classification process, to evaluate if it introduces subjective bias (Estimating the number of errors is dependent on the judgment of the evaluator); (**b**) Hierarchical classification using variables: variables *T_Span_*, *Total_Task1_*, *T_Ratio_*, and *Errors*. Three groups can be clearly differentiated, blue (volunteers 1, 4, 5, 9, 12 and 13), red (volunteers 7, 8, 11 and 14) and green (volunteers 2, 3, 6 and 10); volunteer 10 can be considered either green or red.

Through the video review, it was found, as expected, that the first two exercises were not a challenge for the participants, all of them did exercises one and two without any significant problem. By contrast, the third exercise showed substantial differences between subjects. Table 1 shows results of measurements obtained from video examination. Each row in the table represents the outcomes of a volunteer who did the test. A cluster analysis was performed using two combinations of *T_Span_*, *Total_Task1_*, *T_Ratio_*, and *Errors* (see Table 1); resulting dendrograms are shown in Figure 7. Three groups can be clearly differentiated, blue (volunteers 1, 4, 5, 9, 12 and 13), red (volunteers 7, 8, 11 and 14) and green (volunteers 2, 3, 6 and 10); volunteer 10 can be considered either green or red. Table 2 compares averages and standard deviations for *T_Span_*, *Total_Task1_*, *T_Ratio_*, and *Errors* on each group. As can be seen, participants in the blue group had “better numbers”, as they in general, take less time to finish the exercise, had lower *T_Ratio_* values and made fewer errors. Conversely, the red group had “poorer performance”, as they in general, take long times to finish the exercise, had high *T_Ratio_* values and made many errors. The green group, according to their times, *T_Ratio_* values, quantities of errors, can be considered an “average group”. According to this, volunteers 1, 4, 5, 9, 12 and 13, have higher dexterity; volunteers 2, 3, 6 and 10, have an average level of dexterity; and volunteers 7, 8, 11 and 14, have a low degree of dexterity.

### 3.2. Results from IGlove Data Processing

#### 3.2.1. Exercises Segmentation

**Figure 8:**
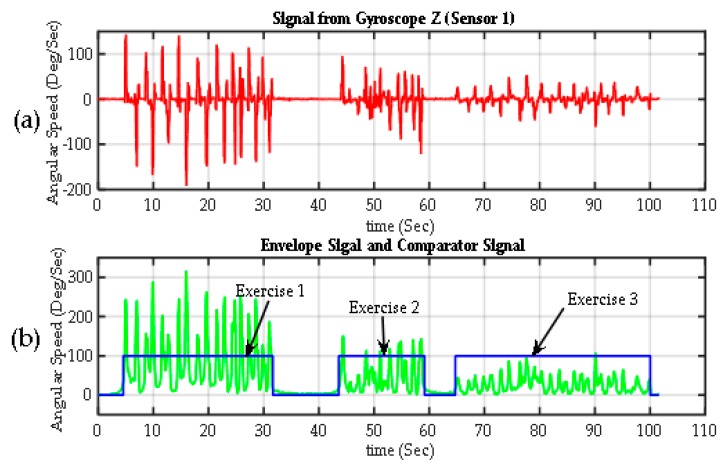
Segmentation of exercises. (**a**) Raw signal from one Z-axis gyroscope from Sensor 1 on the back of the hand ($S_{G1z}$); (**b**) the envelope of the previous signal calculated using the transform Gilbert (green). The exercise detection signal (in blue), which is basically the output of a comparator, with values of 100% when $\left|\hat{S_{G1z}}\right|$ > threshold (exercise is detected) and 0% when $\left|\hat{S_{G1z}}\right|$ < threshold (rest periods).

Figure 8 shows the results of the segmentation algorithm for each of the exercises. The upper graph corresponds to the signal of a gyroscope from one of the participants (Sensor 1, gyroscope on Z-axis),$\;S_{G1z}$ acquired throughout the whole test (the signal from any of the 12 gyroscopes on IGlove could be used). An envelope of this signal$\;\left|\hat{S_{G1z}}\right|$, was calculated using the absolute value of the Hilbert transform of $S_{G1z}$ shown in the second graph (in green), it is a smooth curve that outlines the peaks of $S_{G1z}$ and eliminates most of zero crossings. The envelope signal was passed through a comparator. The comparator output is 100% when $\left|\hat{S_{G1z}}\right|$ is higher than a *Threshold* (the threshold is 15 degrees per second, which is high enough to avoid false activation). If $\left|\hat{S_{G1z}}\right|$ is lower than *Threshold*, the output is set to 0%, indicating a rest period. Small false-positives can be found from time to time in the output signal, mainly caused by small involuntary movements. Also, the output may drop to 0% for short intervals when zero-crossings were found on $\left|\hat{S_{G1z}}\right|$ signal. To overcome these small errors, all peaks in the output of the comparator signal with a duration time of one second or less were eliminated; similarly, all small timeslots (holes) with output at 0% were filled. Table 3 shows the estimated duration time of each exercise.

#### 3.2.2. Movements Segmentation

**Figure 9:**
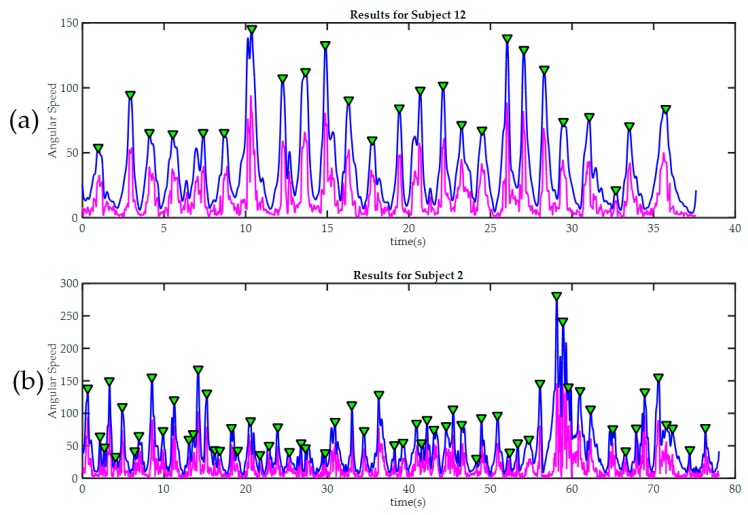
Peak detection results in two trainees during the third exercise. In both cases, the raw signal of magnitude of angular velocity $S_{\omega }$ is painted in magenta. The $S_{\omega }$ signal after being “smoothed” $\left|\hat{S_{\omega }}\right|$ is painted blue. Detected peaks are marked with green triangles. (**a**) Subject 12, required only 25 movements to finish the third exercise. It can be seen how the good level of manual dexterity is also reflected in the in the smoothness of the signal acquired by the sensors in the glove; By contrast, subject 2 (**b**) made many more moves during the exercise; this was because he made several mistakes and then had to correct them. The significant differences in movement amplitude may indicate poor motor control.

Figure 9 Shows results for movement detection in exercise 3 for subjects 12 and 2. Subject 12 was the volunteer with higher hand skills according to video reviewing, and the only one with no errors during this exercise. All peaks detected were marked with green triangles. As previously stated, each peak represents a change in voluntary movements, e.g., in Figure 9a; the second peak marks the instant when the first PB is placed on a peg (instant between *Task1* and *Task2*). Subject 12 required only 25 movements to complete the exercise, other participants who committed errors (like subject 2), required a higher number of movements. Table 4 summarizes results of automatic identification of movements.

#### 3.2.3. Kinematic Analysis Results

**Figure 10:**
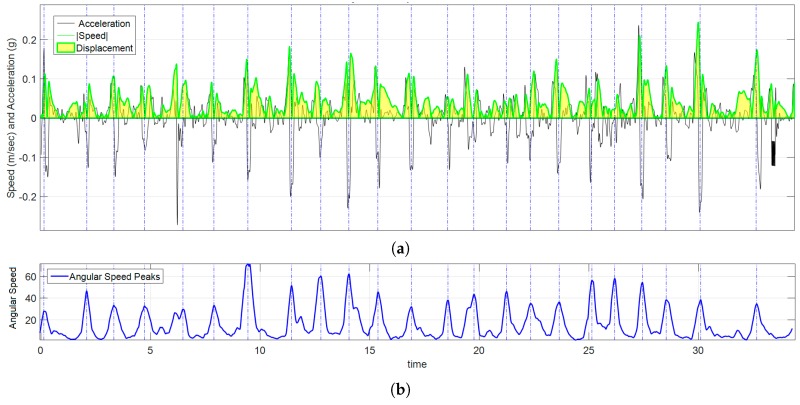
Graph of kinematic spatial parameters for volunteer 12 while playing exercise 3. (**a**) Black signal $S_{a}$ is the magnitude of the acceleration, the absolute value of velocity $\left|V_{a}\left(t\right)\right|$ is drawn in green, and the area under the curve (in clear yellow) is Disp; (**b**) corresponds to the peak detection of $S_{\omega }$, each pair of peaks were used to set the appropriate time interval for numerical calculations.

A signal of the magnitude of the acceleration $S_{a}$ was calculated using Equation (3) from data acquired by accelerometers. By numerical integration of $S_{a}$, the signal of the magnitude of the velocity $V_{a}$ was calculated using Equation (4). The absolute displacement, Disp, i.e., the whole amount of movement made by the volunteer during an exercise is obtained by calculating the area under the curve using Equation (5). An example of this data processing for participant 12 while playing exercise 3 is shown in Figure 10. The total angular displacement during the third exercise was calculated from the signal $S_{\omega }$ using Equation (2). *Disp*, is a measurement of “how much the volunteer moved his/her hand during an exercise”. $Disp_{\theta }$ is a measurement of “how much the volunteer rotated his/her hand during an exercise?” Table 5 shows results of spatial displacement and Table 6 shows results of angular displacement for each volunteer for the three exercises of the IGlove test.

### 3.3. Dimension Reduction Results

IGlove test was divided into three exercises, an exercise can be divided into all movements that a participant made to complete it, and movements were segmented based on $S_{\omega }$ Signal peaks. Table 7 shows the results of a study that focused on the estimation of how similar were movements registered by each of the sensors during the third exercise. To do this, *r* between $S_{\omega }$ signal segments that correspond to each movement was calculated. Subsequently, the average of all these *r* and their standard deviation were calculated. *r* was calculated between sensor 1, located in hand and the other sensors on each finger. Also, the correlation between sensors 2 and 3 is shown to evaluate the activity of thumb-index combination, which is used on tweezers manipulation. Table 8 shows results from the same analysis but for exercises 1 and 2. Table 7 and Table 8, shows *r* calculated between the sensor 1 (in the hand), and the sensors 2, 3 and 4, located on the fingers.

The results for exercises 1 and 3 revealed that in those exercises where *r* is very high, most of the kinematic behavior of the whole hand could be deduced from data acquired on one of the sensors since the information provided by other sensors is redundant regarding a simple measure of cross-correlation. Therefore, analyses regarding FMS were performed using only signals acquired by sensor 1 (extrapolation to other sensors is possible).

**Figure 11:**
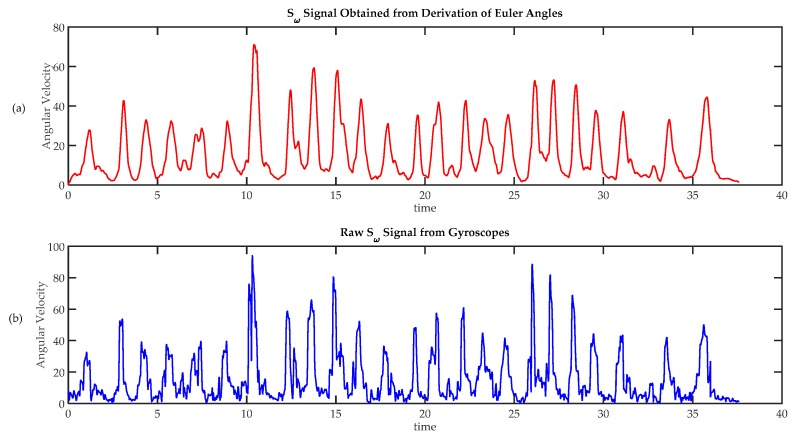
Comparison of techniques to calculate angular speed. (**a**) Angular velocity calculated from absolute orientation angles ϕ, θ and ψ, obtained using a sensor fusion algorithm; (**b**) Angular speed calculated from raw gyroscope data.

Sensor fusion was used to evaluate the utility of including magnetometer signals in the analysis of hand movement. For this purpose, absolute orientation angles ϕ, θ and ψ were calculated based on information from accelerometers, gyroscopes and magnetometers on sensor 1. Angular speeds $\omega_{\phi }$,$\;\omega_{\theta }$ and $\omega_{\psi }$ were calculated using Equation (6). The magnitude of angular velocity $SF_{\omega }$ was calculated using Equation (7). Similarly, the signal of the magnitude of angular velocity $S_{\omega }$ was also calculated using Equation (1) from gyroscopes’ raw data. As an example, Figure 11a shows $SF_{\omega }$ signal and Figure 11b the $S_{\omega }$ signal from data acquired from volunteer 12. As it can be seen, signals were quite similar, the only significant difference is that the first graph is a little bit delayed with respect to the second one (due to a small delay introduced by the fusion algorithm). In this example *r* between $SF_{\omega }$ and $S_{\omega },$ *r* = 0.89, (so they are very similar); these high correlation can also be found in the other exercises, and in all the exercises carried out by the other volunteers (*r* > 0.82 in all cases). So, it was found that $S_{\omega }$ gives basically the same information that $SF_{\omega }$. So, no significant advantages are obtained from sensor fusion; and therefore, signals from magnetometers were not considered. However, in those situations where finger movements have a greater role, information regarding absolute rotation would be useful, this fact will be discussed further on Section 4.

### 3.4. Statistical Analysis Results

**Figure 12:**
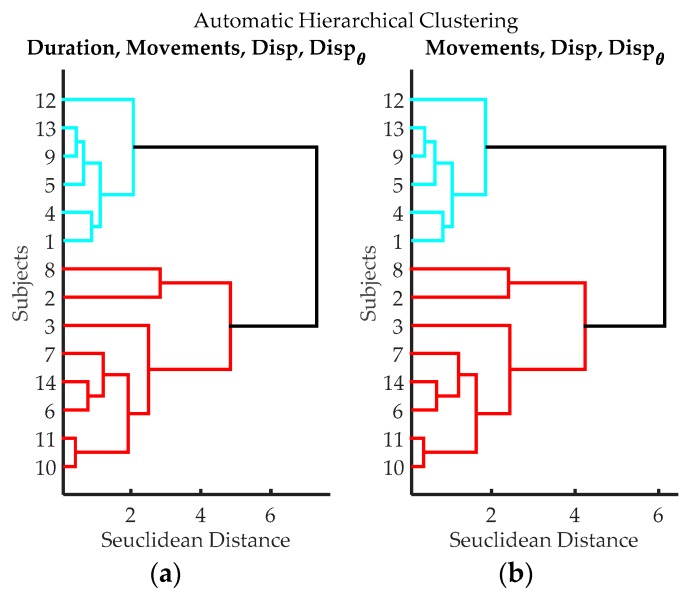
Dendrograms from cluster analysis of volunteers according to variables from data processing of IGlove data. (**a**) Clustering using Time of Exercise, Peaks quantity, spatial displacement and angular displacement; (**b**) Clustering using Peaks quantity, spatial displacement, and angular displacement.

A clustering analysis was made based on the information gathered from IGlove during exercise 3, resulting dendrograms are shown in Figure 12. As a remarkable fact, it can be seen that the algorithm was able to identify the same volunteers with higher FMS that were previously identified in Section 3.1.

Participants were clustered into two groups:

$$\begin{array}{c} Dexterity\;Type\;1=\left\{1,4,5,9,12,13\right\}\\ Dexterity\;Type\;2=\left\{2,3,6,7,8,10,11,14\right\} \end{array}$$

Table 9 shows the average and standard deviation of *Duration*, *Movements*, *Disp* and $Disp_{\theta }$, for both groups, as they can be seen, *Duration* is the variable that better describes the differences between groups, followed by *Movements* and *Disp*. It must be noticed that *Dexterity_Type_1* group corresponds to those volunteers with higher FMS. This result coincides with the result previously found in Section 3.1.

Also, results of statistical validation using Kruskal-Wallis test are presented in Table 10. As can be seen, there are statistical differences between groups in exercise 3, detected by cluster analysis with variables *Duration*, *Movements*, *Disp* and $Disp_{\theta }$. Concerning exercises 1 and 2, statistical result of the test showed that they cannot be used to assess dexterity.

## 4. Discussion

A total of 14 volunteers were recruited to test the IGlove; this quantity may look relatively small, but it should be noted that this population size is consistent with the limited number of residents in most neurosurgery programs [76]. In fact, the neurosurgery program at Universidad de Antioquia (the users of Daubara NS Trainer), admits only three residents per year; there are currently 15 active residents, representing around 15% of all neurosurgical residents in the whole country. To increase the number of potential users of IGlove, and therefore the amount of possible system testers, residents from different medical and surgical specialties should be recruited. Shortly, it is proposed to use IGlove with the residents of general surgery program, who are trained in performing endoscopic procedures using laparoscopic simulators [77].

During the test, rest periods that have been thought to simplify movement recognition process proved to be useful to simplify exercises segmentation, since during these periods the hand remains stationary on the table. However, placing a hand to a specific position to mark the beginning and end of a simulation activity can be impractical for particular types of surgical simulations. As one possible alternative, some authors propose to embed motion sensors inside the surgical instruments [37], in this way, periods of activity can be easily segmented, as they will coincide with those time intervals in which the instrument is being used.

The wrist cuff of the IGlove (i.e., the HUB), is undoubtedly an uncommon element to the traditional surgical practice. However, none of the participants stated that this instrument was uncomfortable. Part of the problem lies in its size because the electronic cards that were used for its implementation were Arduino compatible boards, which results in a rather bulky electronic device. One of the improvements proposed is the design of custom made cards, which will reduce the size of the HUB. However, the HUB cannot be avoided, since an embedded electronic system is required to extract the information acquired by each of the sensors in the glove. Other alternatives, such as providing wireless connectivity, would result in much more bulky sensors that could interfere with the normal hand movement since each sensor would need a wireless transmitter, a battery and a power management system. Another aspect to consider is the length of the cables connecting the sensors to the HUB. One of the alternatives tested was the use of longer cables to move the HUB to the arm. However, it was found that those long lines generated a severe degradation in high-speed digital signals, which forced to reduce their length to about 30 cm. Regardless, the HUB introduces a bias in all subjects, but as the variables are normalized by subject, it is fair to say, that each variable is compared intrasubject which implies that the bias is reduced in all subjects.

The experimental design can be considered useful for surgical simulation, as it was based on the Peg Transfer Board test, which is included in most of the laparoscopic simulators and is widely used for the evaluation of manual dexterity in endoscopic surgery [77]. It is also worth mentioning that such an exercise that involves handling tiny objects using small instruments in a reduced space resembles in many ways to some of the activities that are commonly performed by a surgeon during certain neurosurgical procedures. It was also found that sensors were sensitive enough to detect some kinds of events that may be unnoticeable to any visual evaluator. An example of this type of event is the momentary variation in the speed of rotation of the hand (which can be seen in the $S_{\omega }$ signal like two peaks very close to each other). It was also noticed that some of the volunteers often did unnecessary movements when handling the tweezers (this can be seen on $S_{\omega }$ signal as a long packet of small peaks). While these events were not used in the analysis reported in this paper, it is interesting to denote that these and other “hardly noticeable” events can be registered, and eventually be considered for further analysis.

As mentioned, all volunteers were filmed while they were doing the test. A careful review of video recording was performed to extract a set of metrics to feed an algorithm that clusters volunteers according to the level of manual dexterity that they have. In some sense, this human-driven evaluation can be considered an analogous of the assessment of an expert surgeon on simulated procedures. The Parameters that were shown in Table 1 and can be regarded as both subjective and objective in some ways. In the case of *T_Span_*, *Total_Task1_*, and *T_Ratio_*, they were calculated by estimating the time duration of some activities, so, these could only present a small bias due to possible errors of appreciation by the person in charge of video review. Conversely, *Errors* are highly subjective, as the recognition of mistakes is subject to the personal judgment of the evaluator. The system was able to differentiate individuals according to their level of FMS, regardless of whether *Errors* parameter was taken into account or not; this is a remarkable fact indeed. This behavior can be attributed to the fact that people with higher manual dexterity made fewer mistakes (so their time parameters were better as they had no need to spend more time to correct their mistakes).

Results shown in Table 3 indicate that automatic measurements of the time duration of the third exercise are very similar to those obtained from the video review (column 2 in Table 1), the small differences can be attributed to errors of appreciation during the video review. The Metrics that were used to evaluate manual dexterity are stored in Table 3, Table 4, Table 5 and Table 6, in the columns that correspond to exercise 3. A trend can be seen among them; it is that volunteers with “lower numbers”, i.e., shorter duration of the exercises, fewer movements, and smaller displacement, tend to be the same, as expected, those participants with “lower numbers” were then classified in the group of high FMS. In the second and third columns in Table 4, it can be seen that all participants required almost the same amount of movement to complete exercises one and two. The analysis just mentioned confirms that the first two exercises do not measure any skill from participants (as these exercises were designed to test IGlove’s functionalities). By contrast, the third exercise can be considered a challenging activity where skilled volunteers can finish faster and using fewer movements than their counterparts.

Table 5 and Table 6 show spatial and angular displacements measured during the test. It can be noticed that displacements registered by all of the sensors on IGlove were very similar; this fact is especially noticeable for the case of exercises 1 and 3. Also on Table 7, the high values of the average of *r* and the small variability on them, reveals a strong correlation between movements registered by sensors for all the volunteers, it means that all sensors registered basically the same information during exercise 3. This fact can be attributed to the little difference in finger movements required to manipulate these small tweezers. However, on $r_{23}\left(\sigma_{23}\right)$, a little difference can be found, since $r_{23}$ values were slightly lower and at the same time $\sigma_{23}$ values were a bit higher than the values in the other columns. This variation may evidence the ability of the glove to detect the differences in movement resulting from small tool manipulation. Table 8 shows a similar behavior in the case of exercise 1, where most of the time all movements were very similar (since volunteers were instructed to put the hand on the table for a while just after performing each hand gesture). Only during exercise 2 sensors could register significant differences on movements. The previous results suggest that the information acquired from only one sensor is enough to assess of FMS in the third exercise (as the four sensors are reporting almost the same information).

The combination of the situation above discussed and the fact that it was found that the information from sensor fusion was not relevant for FMS of assessment, might suggest that the problem has been reduced to a point where the capabilities of IGlove are underused. However, such a drastic reduction is valid only for experimental designs where the movements of fingers relative to the hand are small. In those situations, where movements of fingers have a greater role, the information from sensors on the fingers must be taken into account (e.g., when utilizing laparoscopic instruments on the simulation of a minimally invasive surgery). Moreover, several authors had previously used only one sensor to assess manual dexterity [26,78].

As a remarkable fact, it can be said that based on the IGlove it has been possible to estimate some metrics that previously were only available in the most advanced simulators [8,27,79,80]:

1. The duration of each exercise is an important variable since it has been found that people with greater skills and experts can carry out simulation exercises faster. In the surgical field, the ability to perform procedures quickly, significantly reducing patient morbidity [81,82].
2. The number of movements performed is important, since it has been observed that people with greater ability can finish simulation exercises using fewer movements. In the surgical field, skilled surgeons can perform surgical procedures with a significant economy of movement (on real and simulated procedures), if compared to the number of movements (much higher) required by a resident [73,83,84].
3. Both spatial and angular displacement need to be taken into account, as skilled people tend to be able to perform simulation exercises with shorter paths and making fewer turns and rotations than novices [8,9,24]. In the surgical field, and specifically in neurosurgery, best paths are shorter. Surgeons that use optimal paths could reduce the incidence of some injuries caused by fatigue. Also from patient’s point-of-view, shorter paths can reduce injuries to brain tissue that might be due to the unnecessarily large amount of hand movement.

In Section 3.1 the results of the visual evaluation of dexterity were presented, volunteers were divided into groups according to the results given by an expert who reviewed the video records of the third exercise. Similarly, in Section 3.4 the results of the automatic evaluation of dexterity were presented, volunteers were divided into two groups according to the metrics obtained during the third exercise. It can be seen how in both cases, the group of trainees with “better numbers” was the same (subjects 1, 4, 5, 9, 12 and 13). At first glance, it seems that the IGlove could be just as capable as the expert in evaluating FMS. However, a conclusion like this one cannot be made only based on the results obtained with such a small sample of trainees. Larger longitudinal studies are required to corroborate or deny this possibility. Anyway, IGlove has not been conceived as a “replacement of professors”, but as a complementary tool for assessing residents’ FMS during their unsupervised practices.

## 5. Conclusions

Although gloves have been manufactured in different sizes using a thin and elastic fabric they are significantly different from latex or nitrile gloves conventionally used in neurosurgery. This difference in materials may adversely affect the performance of the trainees while training on the simulator. As a possible solution, it is proposed to put IMU sensors over traditional surgical gloves (e.g., using double sided tape), this will give a real tactile sensation to the trainees. Another element of IGlove that can significantly affect the development of the simulations is the HUB since this is not an element that a surgeon will find in an operating room. As previously shown the HUB is necessary for the IGlove to work, nevertheless, the current HUB is bulky so it can affect the performance of the trainee. Accordingly, a redesign of this system is necessary, focused on reducing the size of the HUB to the point where its interference may be considered neglectable.

Through the utilization of the tools described above and probably some new ones, innovative tests that incorporate aspects traditionally explored on Cognitive Neuroscience and Cognitive Psychology can be developed. Some of the mental processes studied by those disciplines include attention, visual memory, spatial perception, coordination, motricity, problem-solving, brain connectivity, among others. Those processes are intrinsically tied to the work of a neurosurgeon in his/her daily work. Most of the research in cognitive sciences is focused on studying children’s intellectual development or evaluating mentally-impaired individuals. However, the implementation of neuropsychological tests for healthy adults, and especially for highly skilled professionals (such as neurosurgeons) remains as an open research field.

An important limitation of this work is that IGlove’s metrics still need to be validated, to do that effectively, a comparison between IGlove’s results and metrics given by standard advanced simulators is necessary. In this regard, in an ongoing study, a group of trainees performs laparoscopic surgery training using a LAPSIM [19] and at the same time they wear an IGlove. The objective is to determine if IGlove metrics are good enough to evaluate the learning curve, compared with LAPSIM metrics which are taken as gold standards. However, the results obtained in this study were not included in this work, as the experiments have not finished yet, and are planned to be published in an upcoming paper.

## Figures and Tables

**Table 1:** Results for each of the volunteers obtained from the review of videos recorded during the third exercise. The Classification column shows resulting hierarchical classification from dendrograms shown in Figure 7.

Volunteer	*T_Span_*	*Total_Task1_*	*T_Ratio_*	Errors
1	42.2	24.9	1.69	2
2	77.7	33.7	2.31	10
3	60.7	33.4	1.82	4
4	48	27.1	1.77	4
5	48.4	21.7	2.23	3
6	61.2	30.9	1.98	6
7	65.1	22.3	2.91	9
8	107.1	25	4.28	17
9	52.6	27.4	1.92	3
10	82.3	28.4	2.9	12
11	74.4	25	2.98	9
12	34.4	18.1	1.9	0
13	46.9	22.9	2.05	6
14	65.6	18.4	3.57	9

**Table 2:** Comparison of the level of dexterity found in each group. Each column represents the average and standard deviations for each variable in each group. The blue group has high dexterity, as they, in general, take shorter times, lower *T_Ratio_* values and made fewer errors. Conversely, the red group has lower dexterity, as they in general, take longer times, higher *T_Ratio_* values and made many errors. The green group has average dexterity.

Group	$\bar{TSpan}\;\left(\sigma_{TSpan}\right)$	$\bar{TotalTask1}\;\left(\sigma_{TotalTask1}\right)$	$\bar{TRatio}\;\left(\sigma_{TRatio}\right)$	$\bar{Errors}\;\left(\sigma_{Errors}\right)$
Blue	45.42 (6.34)	23.68 (3.54)	1.93 (0.19)	3 (2.00)
Green	70.48 (11.16)	31.60 (2.48)	2.25 (0.48)	8 (3.65)
Red	78.05 (19.83)	22.68 (3.12)	3.44 (0.64)	11 (4.00)

**Table 3:** Automatic measurements of the duration of the exercises, time, is represented in seconds.

Volunteer	Exercise 1	Exercise 2	Exercise 3
1	44.0	21.6	43.1
2	33.8	16.3	78.6
3	50.9	29.5	60.7
4	23.6	10.8	47.7
5	40.9	19.5	49.4
6	37.6	19.2	60
7	42.3	29.9	65.1
8	25.4	15.3	106.3
9	46.1	24.5	51.8
10	36.7	17.8	77.9
11	43.1	20.0	74.4
12	28.4	17.3	34.9
13	23.5	13.5	48.3
14	29.5	15.8	66.6

**Table 4:** Automatic detection of movements. Each column shows the number of movements carried out by a volunteer to complete an exercise. Exercises 1 and 2 did not represent any challenge for any of the participants as exercises were carried out with almost the same quantity of movements. On the contrary, the results of the third exercise were very heterogeneous; some participants required few movements to do exercise while others require many more movements. Extra-movements (more than 25) can be attributed to errors or unnecessary moves during the exercise execution.

Volunteer	Exercise 1	Exercise 2	Exercise 3
1	19	11	32
2	19	11	51
3	20	10	35
4	19	10	33
5	19	11	31
6	19	11	47
7	19	10	56
8	19	10	71
9	19	10	32
10	21	10	60
11	20	10	56
12	19	10	25
13	20	10	37
14	19	10	51

**Table 5:** Spatial displacement (*Disp*) measured by all sensors on each of the exercises. E1 means Exercise 1; E2 Exercise 2; and E3 Exercise 3. In all cases, displacements are expressed in meters.

	Sensor 1			Sensor 2			Sensor 3			Sensor 4		
Vol	E1	E2	E3	E1	E2	E3	E1	E2	E3	E1	E2	E3
1	3.63	2.72	1.94	3.91	3.03	2.05	3.67	2.90	1.98	3.84	2.99	2.04
2	5.17	1.34	5.2	5.38	1.59	5.31	5.12	1.49	5.24	5.28	1.60	5.31
3	4.76	1.80	3.16	5.05	2.02	3.29	4.82	1.90	3.21	4.99	1.98	3.26
4	4.41	1.71	2.69	4.65	2.00	2.78	4.38	1.90	2.70	4.58	1.99	2.77
5	3.45	0.86	2.12	3.64	1.13	2.23	3.42	0.98	2.15	3.58	1.10	2.21
6	5.16	2.80	2.27	5.34	3.11	2.38	5.11	2.99	2.31	5.31	3.10	2.37
7	3.92	1.30	2.41	4.22	1.61	2.52	3.94	1.46	2.44	4.13	1.57	2.49
8	1.91	3.12	4.04	2.12	3.41	4.04	1.88	3.27	3.96	2.03	3.38	4.03
9	3.49	3.69	2.35	3.70	4.02	2.40	3.42	3.89	2.34	3.58	3.99	2.40
10	2.91	1.01	3.25	3.18	1.24	3.33	2.92	1.13	3.27	3.11	1.20	3.32
11	3.84	1.87	3.39	4.09	2.10	3.51	3.84	2.00	3.43	4.03	2.10	3.48
12	3.41	1.57	2.02	3.70	1.89	2.07	3.45	1.74	2.00	3.60	1.83	2.06
13	6.27	2.16	2.32	6.51	2.40	2.41	6.27	2.27	2.34	6.42	2.39	2.41
14	2.43	0.97	2.75	2.66	1.19	2.82	2.40	1.09	2.75	2.57	1.19	2.82

**Table 6:** Angular displacement ($Disp_{\theta }$) measured by all sensors on each of the exercises. E1 means Exercise 1; E2 Exercise 2; and E3 Exercise 3. In all cases, angular displacements are expressed in degrees.

	Sensor 1			Sensor 2			Sensor 3			Sensor 4		
Vol	E1	E2	E3	E1	E2	E3	E1	E2	E3	E1	E2	E3
1	1788	515	1387	2234	769	1565	1803	523	1415	2101	727	1568
2	2049	554	2578	2562	879	2854	2043	581	2584	2318	811	2787
3	1413	540	2080	1797	815	2272	1429	558	2052	1601	772	2237
4	1557	731	1432	1925	1104	1598	1561	735	1442	1798	1019	1545
5	1424	456	924	1715	708	1012	1402	478	916	1649	666	991
6	1585	532	1244	1996	795	1368	1575	534	1240	1812	731	1340
7	1530	499	1776	1897	782	1895	1541	523	1764	1734	720	1936
8	1601	666	1869	1980	1000	2032	1584	671	1854	1788	935	2031
9	1586	448	1184	1976	701	1297	1577	464	1198	1835	647	1299
10	1208	515	1415	1532	796	1552	1214	530	1426	1430	745	1559
11	1492	555	1499	1917	862	1714	1519	577	1520	1766	809	1671
12	1795	574	565	2215	892	612	1804	600	557	2085	832	602
13	1734	841	1103	2239	1288	1187	1754	870	1099	2015	1220	1188
14	1445	364	1401	1789	543	1547	1454	369	1418	1655	515	1519

**Table 7:** *r* calculated between sensor 1 and sensors 2, 3 and 4 during exercise 3. Parameter $r_{12}$ is the average of *r* calculated between the signals $S_{\omega 1}$ and $S_{\omega 2}$ over all the movements that each volunteer performed to complete the exercise 3; $\left(\sigma_{12}\right)$ is the standard deviation of these calculations. $r_{13}\left(\sigma_{13}\right)$, gives the same information as previous column, but correlation was performed between sensors 1 and 3. $r_{14}\left(\sigma_{14}\right),$ In the same way as the previous two, brings the information regarding correlation between sensor 1 and 4. Finally, $r_{23}\left(\sigma_{23}\right)$ shows behavior of the combination thumb-index (for exercise 3, it brings information regarding tweezers manipulation).

Exercise 3						
Vol	$r_{12}\left(\sigma_{12}\right)$	$r_{13}\left(\sigma_{13}\right)$	$r_{14}\left(\sigma_{14}\right)$	$r_{23}\left(\sigma_{23}\right)$	$r_{34}\left(\sigma_{34}\right)$	$r_{24}\left(\sigma_{24}\right)$
1	0.90 (0.15)	0.94 (0.10)	0.94 (0.10)	0.89 (0.16)	0.92 (0.17)	0.90 (0.19)
2	0.88 (0.17)	0.92 (0.12)	0.91 (0.14)	0.87 (0.18)	0.94 (0.18)	0.85 (0.20)
3	0.81 (0.10)	0.97 (0.04)	0.96 (0.05)	0.80 (0.11)	0.93 (0.13)	0.83 (0.15)
4	0.87 (0.18)	0.91 (0.16)	0.92 (0.12)	0.85 (0.19)	0.90 (0.14)	0.82 (0.19)
5	0.80 (0.19)	0.90 (0.18)	0.90 (0.17)	0.78 (0.22)	0.89 (0.15)	0.80 (0.21)
6	0.80 (0.20)	0.92 (0.19)	0.91 (0.16)	0.78 (0.23)	0.92 (0.17)	0.81 (0.21)
7	0.88 (0.16)	0.91 (0.13)	0.91 (0.16)	0.86 (0.20)	0.89 (0.16)	0.84 (0.23)
8	0.83 (0.21)	0.88 (0.15)	0.86 (0.19)	0.81 (0.26)	0.91 (0.15)	0.86 (0.22)
9	0.91 (0.11)	0.97 (0.03)	0.98 (0.05)	0.89 (0.12)	0.93 (0.09)	0.91 (0.11)
10	0.80 (0.16)	0.84 (0.27)	0.89 (0.21)	0.78 (0.30)	0.88 (0.21)	0.79 (0.24)
11	0.90 (0.18)	0.91 (0.15)	0.91 (0.15)	0.88 (0.20)	0.89 (0.17)	0.90 (0.21)
12	0.89 (0.18)	0.95 (0.04)	0.95 (0.04)	0.87 (0.18)	0.91 (0.13)	0.85 (0.19)
13	0.85 (0.10)	0.96 (0.06)	0.95 (0.12)	0.85 (0.10)	0.92 (0.11)	0.87 (0.10)
14	0.84 (0.16)	0.89 (0.14)	0.87 (0.21)	0.83 (0.20)	0.90 (0.18)	0.87 (0.21)

**Table 8:** *r* calculated between the sensor 1 and sensors 2, 3 and 4; and between sensors 2 and 3, during exercises 1 and 2. Columns in this table provide the same information shown in Table 7, but in this case for exercises 1 and 2.

	Exercise 1				Exercise 2			
Vol	$r_{12}\left(\sigma_{12}\right)$	$r_{13}\left(\sigma_{13}\right)$	$r_{14}\left(\sigma_{14}\right)$	$r_{23}\left(\sigma_{23}\right)$	$r_{12}\left(\sigma_{12}\right)$	$r_{13}\left(\sigma_{13}\right)$	$r_{14}\left(\sigma_{14}\right)$	$r_{23}\left(\sigma_{23}\right)$
1	0.91(0.12)	0.91(0.12)	0.90(0.17)	0.89(0.14)	0.60(0.24)	0.64(0.26)	0.55(0.24)	0.59(0.28)
2	0.92(0.10)	0.93(0.09)	0.91(0.12)	0.90(0.11)	0.75(0.13)	0.75(0.12)	0.76(0.13)	0.74(0.15)
3	0.84(0.30)	0.81(0.42)	0.86(0.18)	0.79(0.44)	0.76(0.18)	0.77(0.19)	0.80(0.17)	0.75(0.22)
4	0.80(0.13)	0.82(0.13)	0.81(0.14)	0.79(0.14)	0.67(0.18)	0.62(0.20)	0.69(0.18)	0.61(0.25)
5	0.85(0.15)	0.87(0.14)	0.84(0.17)	0.83(0.16)	0.75(0.15)	0.80(0.12)	0.72(0.20)	0.73(0.15)
6	0.90(0.11)	0.91(0.11)	0.90(0.11)	0.89(0.12)	0.67(0.26)	0.65(0.26)	0.67(0.24)	0.65(0.26)
7	0.87(0.15)	0.88(0.14)	0.86(0.15)	0.86(0.16)	0.73(0.21)	0.74(0.18)	0.70(0.18)	0.72(0.24)
8	0.71(0.19)	0.69(0.22)	0.69(0.18)	0.67(0.25)	0.78(0.13)	0.86(0.18)	0.86(0.09)	0.78(0.23)
9	0.91(0.12)	0.90(0.16)	0.92(0.10)	0.88(0.17)	0.77(0.12)	0.86(0.14)	0.85(0.14)	0.76(0.15)
10	0.85(0.21)	0.86(0.21)	0.87(0.20)	0.83(0.26)	0.67(0.21)	0.69(0.18)	0.49(0.30)	0.66(0.26)
11	0.83(0.16)	0.86(0.17)	0.80(0.13)	0.81(0.18)	0.71(0.17)	0.71(0.17)	0.70(0.21)	0.70(0.18)
12	0.92(0.08)	0.94(0.06)	0.91(0.08)	0.89(0.12)	0.67(0.18)	0.75(0.12)	0.68(0.22)	0.66(0.20)
13	0.89(0.17)	0.95(0.04)	0.93(0.10)	0.87(0.20)	0.72(0.26)	0.73(0.22)	0.71(0.26)	0.70(0.34)
14	0.85(0.14)	0.86(0.15)	0.89(0.11)	0.85(0.17)	0.65(0.24)	0.66(0.22)	0.64(0.21)	0.63(0.26)

**Table 9:** Comparison of the results of groups Type 1 and Type 2. Each column represents the average and standard deviations for each variable on both groups. Type 1, has individuals with higher dexterity, as they used less time to complete the exercise, made fewer movements, and had smaller displacements. Type 2, include volunteers who have lower dexterity as the values of parameters were higher.

Group	$\bar{Duration}\;\left(\sigma_{Duration}\right)$	$\bar{Movements}\;\left(\sigma_{Movements}\right)$	$\bar{Disp}\;\left(\sigma_{Disp}\right)$	$\bar{Disp\theta }\;\left(\sigma_{Disp\theta }\right)$
Type 1	45.87 (6.08)	31.67 (3.88)	2.24 (0.27)	1099.17 (321.51)
Type 2	73.70 (15.05)	53.38 (10.41)	3.31 (0.95)	1732.75 (440.78)

**Table 10:** Results from Kruskal-Wallis test for each exercise. In the case of exercises 1 and 2, results of the statistical test show that the IGlove responds in the same way in the case of exercises that are not suitable to assess dexterity. By contrast, the low ρ−values found for exercise 3 indicate a significant difference between groups, therefore, variables *Duration*, *Movements*, *Disp* and $Disp_{\theta }$, acquired during FMS test can be used to assess dexterity using the IGlove.

ρ−Values				
Exercise	Duration	Movements	Disp	$Disp_{\theta }$
Exercise 1	0.6056	0.3705	0.8973	0.1967
Exercise 2	0.6056	0.7420	0.6056	0.6510
Exercise 3	0.0019	0.0029	0.0098	0.0098
